# Response to letter regarding “Developing a predictive model for spinal shock in dogs with spinal cord injury”

**DOI:** 10.1111/jvim.16633

**Published:** 2023-01-27

**Authors:** Rebecca McBride, Elizabeth Parker, Rebecca B. Garabed, Natasha J. Olby, Andrea Tipold, Veronika Maria Stein, Nicolas Granger, Ashley C. Hechler, Page E. Yaxley, Sarah A. Moore, the CANSORT‐SCI study investigators

**Affiliations:** ^1^ Department of Veterinary Clinical Sciences The Ohio State University College of Veterinary Medicine Columbus Ohio USA; ^2^ Department of Veterinary Preventive Medicine The Ohio State University Columbus Ohio USA; ^3^ Department of Clinical Sciences College of Veterinary Medicine Raleigh North Carolina USA; ^4^ Department of Small Animal Medicine and Surgery University of Veterinary Medicine Hannover Germany; ^5^ Department of Clinical Veterinary Sciences, Vetsuisse Faculty University of Bern Bern Switzerland; ^6^ Department of Small Animal Clinical Sciences, School of Veterinary Sciences University of Bristol Bristol UK

Dear Drs Hinchcliff and DiBartola,

Thank you for the opportunity to address the points made by Dr Cummings on our recent paper reporting the development of a clinical predictive model for spinal shock. We thank the author for his thorough review of the literature related to clinical predictive models (CPMs) and agree with his assertion that CPMs must be externally validated before they can be widely deployed for prognostication in the clinical setting. This is a future direction for our group, and a limitation of the current data that we discuss in our manuscript.

Dr Cummings suggests that provision of a simple tool such as a spinal shock calculator would allow others to quickly perform model calculations for their own patients. We would like to point out that, while we did consider this as part of our original work, we felt it would be inappropriate to provide a specific diagnostic tool at this time, given the risk of bias and lack of external validation Dr Cummings carefully points out in his letter.

To address Dr Cummings' concern that dogs with missing data (figure 2) were excluded from the analysis rather than using multiple imputation to estimate missing data, it is relevant to consider that these dogs did not have the basic data in their records that was required to determine their eligibility for the study. This was not just a matter of a missing predictor. It was unclear whether they had either of the outcomes of interest (L4‐S3 myelopathy or T3‐L3 myelopathy with spinal shock) for prediction. Of the 457 dogs with complete data, only 72 had one of the relevant conditions, so for the 61 excluded dogs, if the data were missing at random, only 9 or 10 might have had one of the relevant conditions. Additionally, we were concerned that data might not be missing at random because dogs without the required imaging and examinations could have had conditions that did not require imaging for diagnosis (exclusion criterion). Given that this would both decrease the number of relevant cases excluded and violate the “missing at random” assumption of imputation algorithms, we determined that imputation of missing values would not be appropriate. It is correct that there is a small chance of bias introduced by this choice, but it should be less than what would have been introduced by using multiple imputation.

Regarding Dr Cummings's mention of “training” (n = 64) and “test” (n = 8) data, we would like to clarify that there was no “test” data used in this investigation. We presented hypothetical dogs in Figure 3, Figure 4, and Table 5 to demonstrate clinically relevant examples of the model's prediction. We have found that equations and coefficients can be difficult for clinicians to interpret, so this was simply a different way of demonstrating the model. Apologies if it gave the impression of an attempt at external validation. It was not.

